# Musculoskeletal Tumor Care Disparities Prior to Initiation of Treatment Among Newly Diagnosed Adult Patients

**DOI:** 10.3390/cancers17091519

**Published:** 2025-04-30

**Authors:** Lauren J. Li, Nam Bui, Everett J. Moding, Robert Steffner, David Mohler, Kristen Ganjoo, Minggui Pan

**Affiliations:** 1 Department of Medicine, Division of Oncology, Stanford University, Stanford, CA 94305, USA; laurli@stanford.edu (L.J.L.); nambui@stanford.edu (N.B.); kganjoo@stanford.edu (K.G.); 2 Department of Radiation Oncology, Stanford University, Stanford, CA 94305, USA; emoding@stanford.edu; 3 Department of Musculoskeletal Tumor Service, Stanford University, Stanford, CA 94305, USA; rsteffner@stanford.edu (R.S.); dmohler@stanford.edu (D.M.)

**Keywords:** musculoskeletal tumor, sarcoma, disparity, wait-time, imaging, biopsy, consult

## Abstract

**Purpose:** We investigated care disparities and associated factors along the segments of adult musculoskeletal tumor (MST) care prior to initiation of treatment. **Patients and Methods:** This cohort included newly diagnosed MST patients who were referred to Stanford Medical Center for establishing care from July 2020 to April 2024. We investigated the interval from the onset of symptoms to the first appointment with a primary care provider (PCP wait-time), and the interval from first PCP appointment to obtaining the first imaging study (imaging wait-time) and to obtaining biopsy results (biopsy wait-time). Sarcoma consult wait-time was defined as the interval between referral date and consult date. We performed a survey among sarcoma physicians and non-physician staff on the perception of wait-time. **Results:** Among 402 eligible patients, approximately 38.5% had PCP a wait-time longer than 5 weeks, with young adults and Hispanic patients having the highest rate of such long wait-times. Approximately 20.6% of patients had an imaging wait-time longer than 5 weeks, with young adults having the highest proportion of such long wait-times. In addition, Hispanic (*p* = 0.02), Black (*p* = 0.05) and Caucasian (*p* = 0.02) patients had significantly higher percentages of patients with an imaging wait-time of more than 5 weeks compared to Asians. Approximately 79.3% of patients had a biopsy wait-time longer than 5 weeks, with Black and Hispanic patients having the highest percent of such long wait-times. In addition, compared to public insurance, private insurance was associated with a higher proportion of patients with PCP wait-times, imaging wait-times, sarcoma consult wait-times and biopsy wait-times longer than 5 weeks. The survey responses overwhelmingly indicated that a wait-time of more than 5 weeks was not acceptable. **Conclusions:** Substantial disparities in MST care related to age group, ethnicity and insurance type existed in multiple segments of the care journey prior to the initiation of treatment. Our study provides insights for practice, research and policy considerations for narrowing sarcoma care disparities.

## Simple Summary

Disparities in the care of patients with musculoskeletal tumor (MST) remains not well-understood. We have found that there existed substantial disparities in MST care related to age group, ethnicity, and insurance type in multiple segments of the care journey prior to initiation of treatment. Our study provides insights for practice, research, and policy considerations for narrowing MST care disparities.

## 1. Introduction

There are numerous factors that impact the care quality and disparities of patients with cancer including many social determinants such as age, sex, insurance type, socioeconomic status and geographic location; however, the root cause and the factors associated with the disparities remain not well understood [1,2,3,4]. Treatment delay due to barriers to access to healthcare system and other factors has been found to be associated with worse mortality in several types of cancer [5]. Musculoskeletal tumors (MSTs), including soft tissue and bone sarcomas and certain benign tumors, are uncommon neoplasms yet show vast heterogeneity, causing numerous challenges in their management [6,7,8]. The period from the onset of MST symptoms to completing treatment can often be several months. Potential delays can occur at different segments of the entire journey of the care, including obtaining an appointment with a primary care provider; obtaining imaging studies such as magnetic resonance imaging (MRI), computed topography (CT), positron emission topography scan (PET), and others; obtaining a biopsy of the tumor to establish diagnosis; and obtaining a consultation with a sarcoma oncologist. Delayed access to care in any of these segments of the sarcoma care journey prior to the initiation of treatment could potentially result in inferior outcomes for patients [5]. The delayed access to cancer care during the COVID-19 pandemic has been studied in several fronts and found to be associated with racial disparities and inferior outcomes [5,9].

Multidisciplinary care provided by sarcoma experts is considered critical for optimal outcomes of patients with MST [4,10,11]. In our previous studies, we showed that a virtual multidisciplinary MST case conference platform could potentially enhance care quality and physician confidence [12,13]. There have been several studies that addressed disparities in the care and outcomes of patients with MST. These studies focused on the treatment and outcomes associated with ethnicity, insurance status, rural geography, high MST volume center and others [10,14]. Few studies have focused on the initial segments of MST care prior to the initiation of treatment.

Our study aimed to investigate the potential delays in the initial segments of MST care from the onset of symptoms to the initial visit with a primary care provider, followed by obtaining imaging studies for staging work-up and obtaining biopsies to establish a definitive MST diagnosis, and obtaining an MST consultation to establish care with experts. We found that significant care disparities existed in multiple segments of the initial care journey prior to the commencement of treatment. Our study provides insights into the care gaps and disparity-associated social determinants that could potentially be helpful for research, practice and policy considerations for enhancing the care quality and outcomes of patients with MST.

## 2. Methods

### 2.1. Study Population

Approximately 96% of patients were referred from the community hospitals and had workup performed at the community hospitals outside of Stanford, while approximately 4% of patients were in-network referrals whose workup was performed at Stanford Medical Center. Data including demographics was collected from the electronic medical record (EMR). This study was approved by Stanford’s internal review board.

Inclusion criteria: Eligible patients were 18 years or older with a newly diagnosed MST and were referred to establish care at Stanford Medical Center with an appointment with an adult sarcoma oncologist in the sarcoma oncology clinic from July 2020 to April 2024. Patients could have a diagnosis of high-grade sarcoma, low-grade sarcoma or benign MST including giant cell tumor of bone, tenosynovial giant cell tumor and desmoid tumor.

Exclusion criteria: We excluded patients who had completed part or all of the needed treatments (surgery, radiation, chemotherapy, etc.) prior to the referral to Stanford Medical Center for consultation (including a second opinion) or establishing care. We also excluded patients whose diagnosis was not a musculoskeletal neoplasm.

### 2.2. Definition of High-Grade Sarcoma Versus Other Histology

High-grade sarcoma included undifferentiated pleomorphic sarcoma (UPS), dedifferentiated liposarcoma, pleomorphic liposarcoma, high-grade myxoid liposarcoma, leiomyosarcoma, rhabdomyosarcoma, high- and intermediate-grade myxofibrosarcoma, synovial sarcoma, angiosarcoma, malignant peripheral nerve sheath tumor, epithelioid sarcoma, epithelioid hemangioendothelioma, high-grade endometrial stroma sarcoma, radiation-induced sarcoma, unspecified high-grade sarcoma, osteogenic osteosarcoma, Ewing sarcoma, high-grade and dedifferentiated chondrosarcoma, etc. The Other category included well-differentiated liposarcoma, low-grade myxoid liposarcoma, gastrointestinal stromal tumor (GIST), perivascular epithelioid cell tumor, solitary fibrous tumor, low-grade fibromyxoid sarcoma, low-grade myxofibrosarcoma, follicular dendritic cell sarcoma, inflammatory myofibroblastic tumor, Kaposi’s sarcoma, low-grade endometrial stroma sarcoma, chordoma, phyllodes tumor, low- and intermediate-grade conventional chondrosarcoma, as well as benign histology including giant cell tumor of bone, tenosynovial giant cell tumor, desmoid tumor, leiomyomatosis, and others.

### 2.3. Definition of Segments of MST Care Prior to Initiation of Treatment

**Figure 1:**
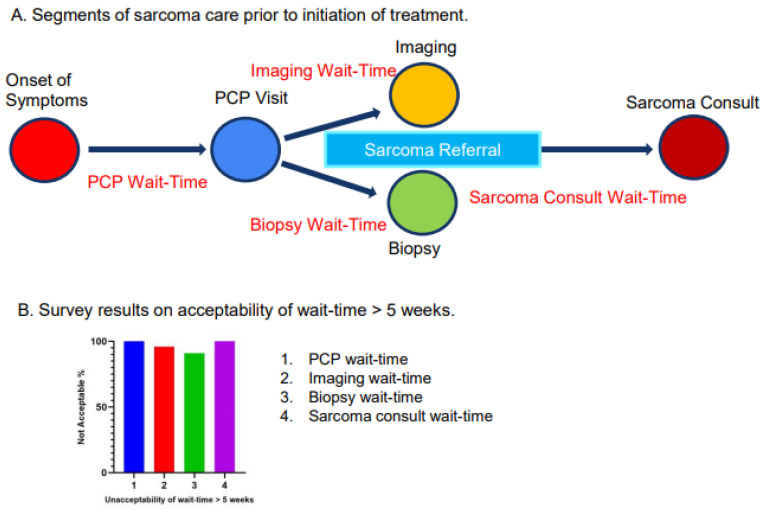
(**A**). Illustration of the segments of MST care prior to initiation of treatment. The interval from the onset of MST symptoms (pain and/or a palpable mass) to the initial visit with a primary care provider (PCP) was defined as PCP wait-time. The interval from the initial PCP visit to obtaining the first imaging study (MRI, PET/CT or CT scan, etc.) was defined as imaging wait-time. The interval from the PCP visit to the biopsy date was defined as biopsy wait-time. The interval between the sarcoma referral date and the consult date was defined as sarcoma consult wait-time. (**B**). Survey results on acceptability of wait-time >5 weeks. Questionnaires included the following: 1. Is waiting for more than 5 weeks to see a Primary Care Provider (PCP) after the onset of sarcoma symptoms (pain and/or palpable mass) acceptable? 2. Is waiting for more than 5 weeks to obtain an Imaging Study (MRI, CT, or PET/CT, etc.) acceptable? 3. Is waiting for more than 5 weeks to obtain a Biopsy acceptable? 4. Is waiting for more than 5 weeks to see Sarcoma Oncologist for Consult acceptable? The options for response were “Yes” or “No”.

Figure 1A illustrates the definition of wait-time in different segments. The interval from the onset of MST symptoms (pain and/or a palpable mass) to the initial visit with a primary care provider (PCP) was defined as PCP wait-time. The interval from the initial PCP visit to obtaining the first imaging study (MRI, PET/CT or CT scan, etc.) was defined as imaging wait-time. The interval from the PCP visit to the biopsy date was defined as biopsy wait-time. The interval between the sarcoma referral date and the consult date was defined as sarcoma consult wait-time.

### 2.4. Insurance Type

Medicare and MediCal were defined as public insurance while commercial insurances were defined as private insurance.

### 2.5. Distances from Stanford Medical Center

Distance from Stanford Medical Center was calculated based on the patient’s home address in miles. It primarily helps to understand if patients were from metropolitan versus non-metropolitan areas. Those who lived 50–75 miles or farther away from Stanford Medical Center were likely living in a non-metropolitan area.

### 2.6. Survey Questionnaires

We conducted a survey within the Stanford sarcoma program among physicians and non-physician staff to understand the perception of the acceptability of wait-times of more than 5 weeks. The responders included medical oncologists, radiation oncologists, pathologists, radiologists, pediatric oncologists, surgical and musculoskeletal oncologists, nurse practitioners, nurse care coordinators, clinical trial coordinators and medical assistants. The four questions posted were the following: 1. Is waiting for more than 5 weeks to see a Primary Care Provider (PCP) after the onset of sarcoma symptoms (pain and/or palpable mass) acceptable? 2. Is waiting for more than 5 weeks to obtain an Imaging Study (MRI, CT, or PET/CT, ect.) acceptable? 3. Is waiting for more than 5 weeks to obtain a Biopsy acceptable? 4. Is waiting for more than 5 weeks to see Sarcoma Oncologist for Consult acceptable? The options for response were “Yes” or “No”.

### 2.7. Statistical Analysis

We used descriptive statistics to describe the percentage of patients who had wait-times of within 2 weeks (1–2 weeks), 3–5 weeks and more than 5 weeks, according to their differences in age group, ethnicity, distance, insurance type, histology and sex. We used chi-square tests to compare the statistical differences between different subgroups.

## 3. Results

### 3.1. Demographics

The percentages of male and female patients were roughly equal (50.25% versus 49.75%). Approximately 42.0% of patients had public insurance (Medicare or MediCal; n = 169) and 58.0% had private insurance (n = 233). Approximately 52.2% of patients had high-grade sarcoma versus 47.8% with Other histology. Approximately 9.9%, 23.4%, 34.6% and 32.1% of patients were young adults (age 18 to 30 years old), aged 31–50 years old, aged 51 to 70 years old and aged older than 70 years. Approximately 54.2% of patients were Caucasian (n = 218), 20.6% were Asian (n = 83), 3.2% were Black (n = 13) and 16.4% were Hispanic (n = 66). Approximately 29.3%, 16.4%, 8.0%, 10.7% and 35.6% of patients lived within 30 miles, 31–50 miles, 51–75 miles, 76–100 miles and farther than 100 miles from Stanford Medical Center (Table 1).

### 3.2. Survey Results

We performed a survey among physicians and non-physicians within the Stanford sarcoma program to assess their perception of the acceptability of wait-times of more than 5 weeks (Figure 1B). There were 23 responses received. On question 1 (Is waiting for more than 5 weeks to see PCP after the onset of sarcoma symptoms acceptable?), 100% of responses were “No”; on question 2 (Is waiting for more than 5 weeks to obtain an Imaging Study acceptable?), approximately 96% of responses were “No”; on question 3 (Is waiting for more than 5 weeks to obtain a Biopsy acceptable?), approximately 93% of responses were “No”; and on question 4 (Is waiting for more than 5 weeks to see Sarcoma Oncologist for Consult acceptable?), 100% of responses were “No”.

### 3.3. PCP Wait-Time

**Figure 2:**
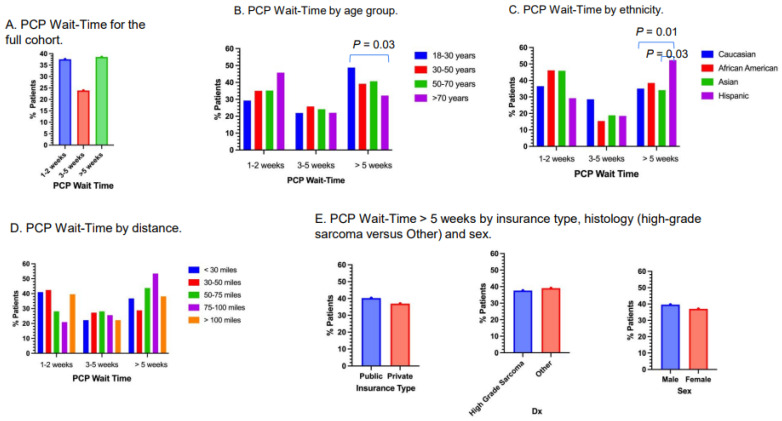
PCP wait-time. (**A**) PCP wait-time of the full cohort. (**B**), PCP wait-time by age group. Young adults (age 18–30 years) had a significantly high proportion of patients with wait-times of more than 5 weeks compared to patients older than 70 years (*p* = 0.03). (**C**) PCP wait-time by ethnicity. Hispanic patients had significantly higher proportion of patients with wait-times of more than 5 weeks compared to Caucasians (*p* = 0.01) and Asians (*p* = 0.03). (**D**) PCP wait-time by distance. Distances were measured from patients’ residence to Stanford Medical Center. (**E**) PCP wait-times of more than 5 weeks by insurance type, high-grade or Other, and sex.

Approximately 37.6% of patients had PCP wait-times of 1–2 weeks, 23.9% had PCP wait-times of 3–5 weeks and 38.5% had PCP wait-times of more than 5 weeks (Figure 2A). Approximately 48.9% of young adults (age 18 to 30 years old) had PCP wait-times of more than 5 weeks, versus 38.1% of patients 31–50 years old, 40.7% of patients 51–70 years old and 32.2% of patients older than 70 years (Figure 2B). The difference between the young adult group and patients older than 70 years old was statistically significant (*p* = 0.04). Approximately 52.3% of Hispanic patients had PCP wait-times of more than 5 weeks, versus 35.0% for Caucasians (*p* = 0.01), 38.5% for Black patients (*p* = 0.36) and 34.1% for Asians (*p* = 0.03; Figure 2C). Patients who lived 71–100 miles from Stanford Medical Center appeared to have the highest rate of PCP wait-times of more than 5 weeks; however, there was no apparent pattern regarding distance in relation to PCP wait-time (Figure 2D). Approximately 40.2% of patients with private insurance (n = 93) had PCP wait-times of more than 5 weeks, versus 36.9% (n = 61) for patients with public insurance. Approximately 37.6% of patients with high-grade sarcoma had wait-times of more than 5 weeks versus 39.0% of Other histology. Approximately 39.6% of male patients had PCP wait-times of more than 5 weeks, versus 37.0% of female patients (Figure 2E).

### 3.4. Imaging Wait-Time

**Figure 3:**
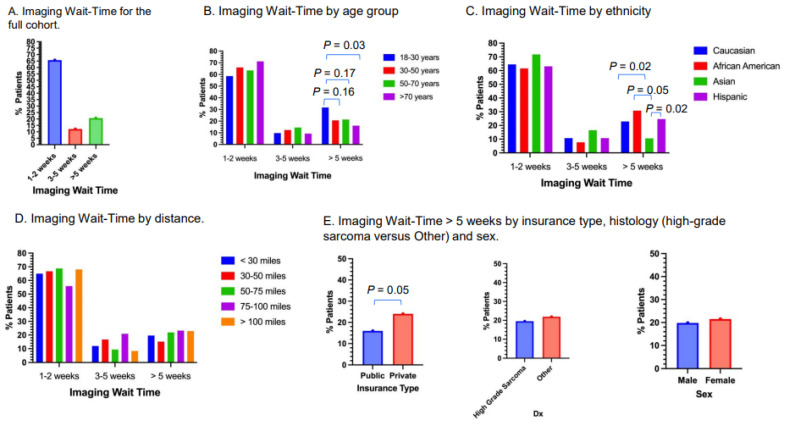
Imaging wait-time. (**A**) Imaging wait-time of the full cohort. (**B**) Imaging wait-time by age group. Young adults (age 18–30 years) had a significantly high proportion of patients with wait-times of more than 5 weeks compared to patients older than 70 years (*p* = 0.03). (**C**) Imaging wait-time by ethnicity. Hispanic (*p* = 0.02), Black (*p* = 0.05) and Caucasian (*p* = 0.02) patients had significantly higher proportions of patients with wait-times of more than 5 weeks compared to Asian patients. (**D**) Imaging wait-time by distance. Distances were measured from patient residence to Stanford Medical Center. (**E**) Imaging wait-times of more than 5 weeks by insurance type, high-grade or Other, and sex. A significantly higher proportion of patients with private insurance had wait-times of more than 5 weeks compared to patients with public insurance (*p* = 0.05).

Approximately 65.7% of patients had imaging wait-times of within 1–2 weeks, 12.2% had imaging wait-times of 3–5 weeks and 20.6% had imaging wait-times of more than 5 weeks (Figure 3A). Approximately 31.7% of young adults had imaging wait-times of more than 5 weeks, compared to approximately 20.6% of patients 31–50 years old (*p* = 0.16), 21.3% of patients 51–70 years old (*p* = 0.17) and 16.1% of patients older than 70 years (*p* = 0.03; Figure 3B). Approximately 30.8% of Black patients had imaging wait-times of more than 5 weeks, in addition to 22.9% for Caucasian, 10.6% for Asian and 24.6% for Hispanic patients (Figure 3C). Compared to Asian patients, Hispanic (*p* = 0.02), Black (*p* = 0.05) and Caucasian (*p* = 0.02) patients had significantly higher rates of imaging wait-times of more than 5 weeks. There were no apparent differences in imaging wait-time among the different distances from Stanford Medical Center (Figure 3D). Approximately 24.0% of patients with private insurance had imaging wait-times of more than 5 weeks, versus 16.0% for patients with public insurance (*p* = 0.05; Figure 3E). Approximately 19.5% of patients with high-grade sarcoma and 21.9% of Other histology had wait-times of more than 5 weeks (Figure 3E). Approximately 19.8% of male and 21.5% of female patients had imaging wait-times of more than 5 weeks (Figure 3E).

### 3.5. Sarcoma Consult Wait-Time

**Figure 4:**
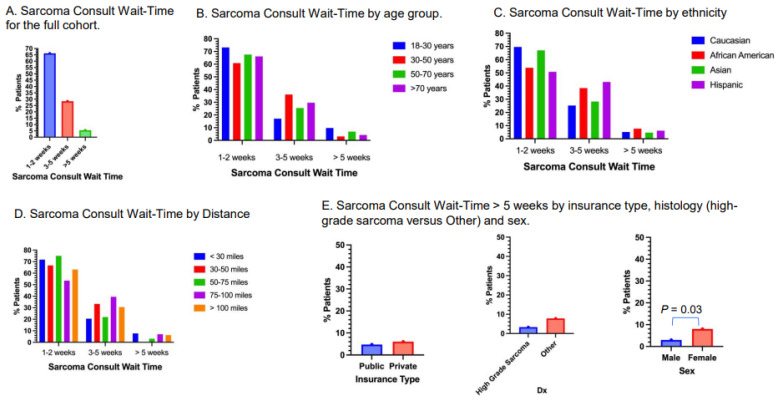
Sarcoma consult wait-time. (**A**) Sarcoma consult wait-time of the full cohort. (**B**) Sarcoma consult wait-time by age group. (**C**) Sarcoma consult wait-time by ethnicity. (**D**) Sarcoma consult wait-time by distance. (**E**) Sarcoma consult wait-time of more than 5 weeks by insurance type, high-grade or Other, and sex. A significantly higher proportion of female patients had wait-times of more than 5 weeks compared to male patients (*p* = 0.03).

Approximately 66.2% of patients had sarcoma consult wait-times of 1–2 weeks, 28.4% had sarcoma consult wait-times of 3–5 weeks and 5.5% had sarcoma consult wait-times of more than 5 weeks (Figure 4A). The sample size for patients with sarcoma consult wait-times of more than 5 weeks is small for all subgroups. Approximately 9.8% of young adults had sarcoma consult wait-times of more than 5 weeks, versus 3.1% for patients 31–50 years old, 6.9% for patients 51–70 years old and 4.2% for patients older than 70 years old (Figure 4B). All ethnicities, distances from Stanford Medical Center, insurance types and histology types had 7% or less sarcoma consult wait-times of more than 5 weeks (Figure 4C–E). Approximately 8.0% of female patients had sarcoma consult wait-times of more than 5 weeks, versus 3.0% of male patients (*p* = 0.03; Figure 4E).

### 3.6. Biopsy Wait-Time

**Figure 5:**
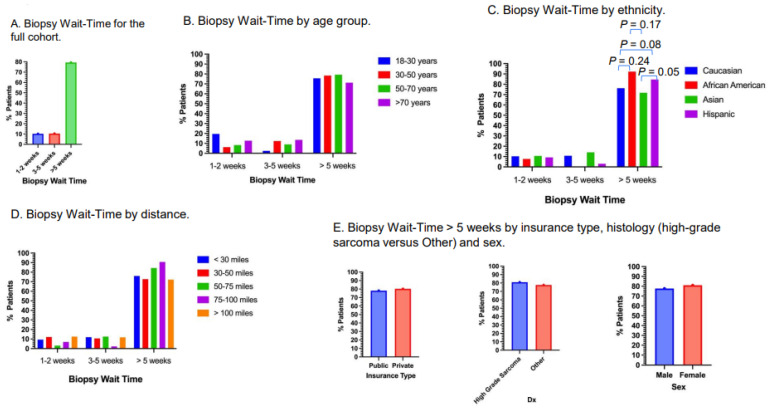
Biopsy wait-time. (**A**) Biopsy wait-time of the full cohort. Nearly 80% of all patients had wait-times of more than 5 weeks. (**B**) Biopsy wait-time by age group. No significant differences among age groups. (**C**). Biopsy wait-time by ethnicity. Hispanic patients had significantly higher proportion of patients with wait-time more than 5 weeks compared to Asians (*p* = 0.05) and borderline significantly higher proportion of patients with wait-time more than 5 weeks compared to Caucasians (*p* = 0.08). (**D**) Biopsy wait-time by distances. Distances were measured from patient residence to Stanford Medical Center. (**E**). Biopsy wait-times of more than 5 weeks by insurance type, high-grade or Other, and sex.

Approximately 10.2% of patients had biopsy wait-times of within 1–2 weeks, 10.4% had biopsy wait-times of 3–5 weeks and 79.3% had biopsy wait-times of more than 5 weeks (Figure 5A). Approximately 80.5% of young adults had biopsy wait-times of more than 5 weeks, 81.4% for patients 31–50 years old, 82.8% for patients 51–70 years old and 73.7% for patients older than 70 years old (Figure 5B). Approximately 92.3% of Black patients had wait times of more than 5 weeks versus 79.0% for Caucasian (*p* = 0.10), 75.3% for Asian (*p* = 0.07) and 87.7% for Hispanic patients (Figure 5C). Compared to Asians, Hispanic patients had a significantly higher rate of biopsy wait-times of more than 5 weeks (*p* = 0.05; Figure 5C). Patients who lived 71–100 miles from Stanford Medical Center appeared to have the highest rate of biopsy wait-times of more than 5 weeks, but there was no clear pattern of correlation for the distances (Figure 5D). Approximately 78.1% of patients with public insurance versus 82.3% with private insurance had biopsy wait-times of more than 5 weeks (Figure 5E). Approximately 80.9% of patients with high-grade sarcoma and 77.6% of Other histology had wait-times of more than 5 weeks (Figure 5E). Approximately 77.7% of male and 81.0% of female patients had biopsy wait-times of more than 5 weeks (Figure 5E).

## 4. Discussion

In this study, we utilized survey results and analysis of the retrospective data from Stanford Medical Center, a major tertiary MST care center in Northern California, to investigate the MST care disparities along the segments of care prior to the initiation of treatment. The survey responses overwhelmingly indicated that PCP wait-times, imaging wait-times, sarcoma consult wait-times as well as biopsy wait-times of more than 5 weeks were not acceptable. Unfortunately, nearly 40% of newly diagnosed MST patients had PCP wait-times of more than 5 weeks, and among them young adults had significantly higher rates of such long wait-times compared to patients older than 70 years, and Hispanic patients had significantly higher proportion of such long wait-times compared to Caucasian and Asian patients. In addition, approximately 21% of patients had imaging wait-times of more than 5 weeks, and among them young adults had a significantly higher proportion of such a long wait-time, and Black and Hispanic patients appeared to have higher proportions of such a long wait-time compared to Caucasian and Asian patients. Moreover, there was a significantly higher proportion of patients with private insurance who had such a long wait-time compared to patients with public insurance. The proportion of long wait-times of more than 5 weeks for sarcoma consult was approximately 5% only, and female patients had a significantly higher proportion of such a long wait-time compared to male patients, despite the small sample size. Most strikingly, approximately 80% of patients had a biopsy wait-time of more than 5 weeks and Black and Hispanic patients appeared to have a higher proportion of such a long wait-time compared to Caucasian and Asian patients. In addition, nearly 80% of patients with high-grade sarcoma had biopsy wait-times of more than 5 weeks, similar to the patients who had low-grade or benign musculoskeletal neoplasm that are associated with more indolent disease and favorable outcomes.

The management of MST is particularly complex due to its rarity and heterogeneity and requires multidisciplinary expertise and collaboration [6,7]. The disparity in the care access, quality and outcomes of sarcoma have long been a concern to the cancer community, from misdiagnosis to delay in diagnosis and survival outcomes [4,14,15,16]. Multidisciplinary care including utilizing telemedicine has been proposed to improve access, care quality and ultimately outcomes, especially for patients who live in suburban areas [14,17]. Our previous studies within the Kaiser Permanente system had demonstrated the value of a virtual multidisciplinary sarcoma case conference [12,18]. The NETSARC study (funded by the French National Cancer Institute) showed that a multidisciplinary sarcoma tumor board was associated with improved compliance with practice guidelines and relapse-free survival [19]. This type of multidisciplinary and collaborative platform takes the advantages of care systems such as Kaiser Permanente and the Canadian and French healthcare systems; however, it is not clear how applicable such a platform could be outside of a management or national healthcare system.

There have been several other studies that have shown disparities in MST care and outcomes. Lazarides et al. studied the National Cancer Database (NCDB) and found that Black patients had high Charlson comorbidity index, poorer resources, a higher rate of amputation and worse survival compared to Caucasian and Asian patients [20]. A study using the Surveillance, Epidemiology, and End Results (SEER) database with more than 7000 patients showed that Black patients had more advanced stage and received fewer resections and less frequent radiation therapy [21]. A study on pediatric sarcoma patients found that Black and Hispanic patients had a higher stage of sarcoma at presentation and worse survival compared to Caucasians and Asians [22]. Similar results were found in adult sarcoma patients in a different study [23]. A study using neighborhood socioeconomical status found that lower socioeconomical status was associated with a lower likelihood of receiving National Comprehensive Cancer Network (NCCN)-recommended radiation therapy and worse disease-specific survival [10,24]. Unfortunately, some sarcomas such as Ewing sarcoma have been found to occur in higher incidence in the Hispanic population and with worse survival [25].

Our study utilized a unique approach and focused on the initial segments of MST care prior to the initiation of treatment to understand the care disparities. Our data confirmed that Black and Hispanic patients endured significant disparities in PCP wait-time, imaging wait-time and biopsy wait-time. These delays in obtaining needed care in the initial segment of sarcoma care prior to the initiation of treatment could conceivably impact patient outcomes by delaying treatment. It has been found that even four weeks of delay in treatment initiation could be associated with increased mortality in several types of cancer including head and neck cancer and others [5,26,27]. For example, a prolonged wait-time for radical cystectomy after transurethral bladder resection was shown to be associated with inferior overall survival (OS) for patients with bladder cancer [28]. A longer wait-time to surgery for breast cancer was found to be associated with worse OS [29]. A large SEER data study with more than ninety-thousand patients showed that OS was approximately 10% worse for every 30 days of delay to surgery for stage I and II breast cancer patients [30].

In addition, we found that young adults had a significantly higher proportion of long PCP and imaging wait-times. This could be related to the possibility that most of the young adults did not have an established primary care provider prior to the onset of symptoms compared to the older adults. This calls for enhanced attention on integrating young adults into the healthcare system as soon as they begin to seek medical attention to reduce delays in care. Previous studies have suggested that OS for young adults has not improved as much as in children and old adults in several cancer types over the past three decades [31,32]. A study on a cohort of patients with early-stage synovial sarcoma found that patients who presented with pain had longer wait-times before seeking medical attention, possibly due to pain being a nonspecific symptom, which led to delays in establishing the diagnosis [33,34]. In our current study, we also found that there was a significantly higher proportion of patients with private insurance with imaging wait-times of more than 5 weeks, and it is not clear what factors were associated with such a finding; one factor could be due to the long process of obtaining an insurance authorization. Our finding that female patients had a significantly higher proportion of sarcoma consult wait-times of more than 5 weeks, despite the small sample size, raises questions on potential gender disparity, for which a survival difference was recently shown in those with advanced intermediate- to high-grade soft tissue sarcoma [35]. Studies have found that delayed access to care is associated with advanced stages in several types of sarcoma at presentation [36]. A study from Royal Orthopedic Hospital in the United Kingdom suggested that compliance with the referral guidelines could be related to delays [37]. A single institution study suggested that provisional diagnosis by radiology may be influential in the length of delays [38]. The complexity of sarcoma diagnosis is likely higher than other types of malignancy, as recognized by an Australian study which proposed establishing sarcoma diagnostic pathways and educational curricula [39]. The lack of recognition of sarcoma symptoms amongst providers can be a factor in delays. The period of our study overlapped with the COVID-19 pandemic, which might have affected the results, as a previous study had shown inequity in cancer care during the pandemic [9]. Reducing delays in access to care could conceivably improve the outcomes of sarcoma patients, including decreasing mortality and the rate of amputation.

Our study has limitations. We did not have the socioeconomic, education or income data to further dissect the factors associated with the long wait-times in different segments of MST care. Our distance data could only be used to estimate whether a patient lived within a metropolitan area or not and could not pinpoint the exact relation between their residence and their local care facility. In addition, this was an observational study and did not obtain data on the cancer stage or survival outcomes to correlate the wait-times and outcomes.

Our study also has several strengths. We dissected the initial journey of MST care prior to the initiation of treatment and investigated the proportion of long wait-times of more than 5 weeks among different groups in terms of age, ethnicity, metropolitan or non-metropolitan area, sex, insurance type and sarcoma histology. This approach provides a mechanism to obtain deeper insight on the delayed access to care than what has been previously reported.

In conclusion, our study focused on the initial segments of MST care prior to the initiation of treatment and revealed care disparities associated with age group, ethnicity, insurance type and sex. Our data provide new insights for practice, research and policy considerations in enhancing care access, improving quality and narrowing disparities.

## Figures and Tables

**Table 1:** Demographics of the full cohort. Public insurance included Medicare and MediCal. The distances were measured from patients’ residence to Stanford Medical Center and were suggestive of metropolitan versus non-metropolitan areas (patients who live farther than 50–75 miles were likely in a non-metropolitan area).

		All Patients (N = 402)	Percentage of All Patients (%)
Sex			
Male		202	50.25
Female		200	49.75
Insurance Type			
Public		169	42.0
Private		233	58.0
Histology			
High-grade		210	52.2
Other	Low-grade	136	33.8
	Benign	56	13.9
Age (years)			
18–30		40	9.9
31–50		94	23.4
51–70		139	34.6
>70		129	32.1
Ethnicity			
Caucasians		218	54.2
Asian		83	20.6
Black		13	3.2
Hispanic		66	16.4
Other		22	5.5
Distance (miles)			
<30		118	29.3
31–50		66	16.4
51–75		32	8.0
76–100		43	10.7
>100		143	35.6

## Data Availability

Stanford University Institutional Review Board has not provided approval for the data on individual patients used in this study to be placed in a public access repository. However, researchers can request access to use this study data by contacting corresponding author.
